# Breed-Specific Responses of Rabbit Semen to Chilling Storage: Sperm Quality, Acrosome Status, and Oxidative Stress Biomarkers

**DOI:** 10.3390/ani15162384

**Published:** 2025-08-14

**Authors:** Ibtissem Boulbina, Mohammed El Amine Bekara, Hacina AinBaziz, Simona Mattioli, Cesare Castellini

**Affiliations:** 1 Laboratory of Research “Health and Animal Productions”, Higher National Veterinary School “Rabie Bouchama”, Issad Abbes Street, Oued Smar, Algiers 16000, Algeria; h.ainbaziz@ensv.dz; 2 Laboratory of Molecular Biology, Genomics and Bioinformatics, Department of Biology, Faculty of Nature and Life Sciences, University Hassiba Benbouali of Chlef, Chlef 02000, Algeria; m.bekara@univ-chlef.dz; 3 Department of Agricultural, Food and Environmental Science, University of Perugia, Borgo XX Giugno 74, 06124 Perugia, Italy; simona.mattioli@unipg.it (S.M.); cesare.castellini@unipg.it (C.C.)

**Keywords:** chilled semen storage, sperm quality, acrosome status, seminal plasma, oxidative stress, antioxidant enzymes, rabbit

## Abstract

Artificial insemination (AI) in rabbits depends largely on chilled semen storage, but the physiological responses to chilling and associated biochemical changes in seminal plasma (SP) remain poorly understood, particularly across breeds. This study aimed to compare the semen preservation capacity of Algerian local population (LAP) and New Zealand White (NZW) rabbits and to explore the relationship between SP oxidative stress biomarkers and sperm traits during 72 h of chilled storage at 5 °C. Semen pools (nine/breed) were evaluated at 0, 4, 24, 48, and 72 h for motility, viability, and acrosome status. Oxidative stress markers were also assessed in the SP, including malondialdehyde (MDA), reactive oxygen metabolites (ROMs), superoxide dismutase (SOD), glutathione peroxidase (GPX), and catalase (CAT). LAP sperm showed higher motility (*p* < 0.001) and viability (*p* < 0.05), particularly between 4 h and 48 h, and exhibited a lower rate of acrosome reaction (*p* < 0.001) from 48 h to 72 h. Lower SOD and higher CAT activity in LAP (*p* < 0.001), correlated with MDA and acrosome status, respectively, may reflect a more balanced antioxidant response. Lipid peroxidation did not appear to be the main factor driving sperm deterioration (*p* > 0.05). These results demonstrate that LAP rabbits exhibit better resilience to chilled storage compared to NZW and highlight the potential value of CAT and SOD activities as indicators of sperm resilience during chilled storage. Further studies are required to validate and extend these findings, with the aim of improving semen preservation strategies.

## Simple Summary

Artificial insemination is commonly used in rabbit breeding, and its success depends on the ability to store semen at low temperatures without damaging the sperm. However, we still do not fully understand how different rabbit breeds cope with cold stress or how the surrounding fluid, known as seminal plasma, helps protect the sperm. In this study, we compared semen from the local Algerian rabbit population and the widely used New Zealand White breed during three days of cold storage. We assessed sperm quality alongside markers of stress and antioxidant activity in the seminal plasma. The local Algerian rabbits tended to maintain better sperm quality during the early stages of storage and showed differences in antioxidant enzyme activity, which may be linked to their resilience. Moreover, a common form of cell damage, called lipid peroxidation, was not the main reason for the decline in sperm quality over time. These findings provide new insights into breed-related differences in rabbit semen preservation and may suggest the potential value of some antioxidant enzymes in assessing sperm resilience during cold storage.

## 1. Introduction

Artificial insemination (AI) is the most widely used reproductive biotechnology across livestock species and is a key tool for genetic and production improvement [1]. In rabbits, AI is widely used in commercial systems and primarily relies on high-performance breeds such as the New Zealand White (NZW) and Californian, which have been selectively bred for their superior reproductive and productive performance [2]. These breeds dominate intensive production systems, with NZW and its derived synthetic lines commonly used as standard paternal lines due to their well-established reproductive traits, consistent semen quality, and adaptability to controlled farming environments [3].

Despite the widespread use of these standardized breeds, AI in rabbits still typically relies on freshly diluted semen used within 12 h of collection, limiting its use to short-distance transport [4]. Extending semen viability beyond this time window would support broader-scale AI programs and reduce logistical constraints, particularly in resource-limited settings. To overcome this constraint, chilled semen storage has emerged as a simple and cost-effective alternative [5]. However, maintaining sperm quality beyond 48 h remains a challenge, as chilling stress leads to a progressive decline in motility, viability, and, ultimately, fertilizing capacity [4,6,7]. The improvement in semen preservation is limited by a lack of fundamental knowledge about the physiological and biochemical changes that sperm undergo during handling and storage [8].

The role of oxidative stress (OS) in the male reproductive function has recently gained considerable attention due to its detrimental effects on semen quality and fertility [9]. During sperm preparation, cooling, and preservation processes, OS, an imbalance in the production and scavenging of reactive oxygen and nitrogen species (RONS), has been considered one of the primary factors of sperm damage [10,11]. These reactive molecules, such as superoxide anions (O_2_•−), hydrogen peroxide (H_2_O_2_), and nitric oxide (•NO), are primarily produced by leucocytes and dead, abnormal, or immature spermatozoa. In addition, spermatozoa themselves produce RONS as a natural byproduct of oxidative metabolism [10,12,13]. At optimal concentrations, RONS play several important roles in sperm physiology [12,13]. However, exposure of sperm to a stressful environment during storage is associated with excessive RONS accumulation, inducing lipid peroxidation (LPO), protein oxidation, mitochondrial dysfunction, and DNA fragmentation, ultimately leading to sperm cell death and a decline in fertility [12,13,14,15]. To maintain RONS homeostasis, semen is endogenously provided with complex antioxidant defense systems in both sperm and seminal plasma (SP). However, the relatively low amount of intracellular antioxidants makes SP the primary line of defense against OS [16].

The evaluation of OS markers and their relationship with semen quality is crucial for understanding sperm physiology and improving semen preservation for successful AI. Nevertheless, there is limited information available concerning the relationship between the rabbit semen response to storage and OS markers. In particular, the literature remains scarce regarding the role of endogenous antioxidants in protecting rabbit sperm from oxidative damage. Most existing studies on OS in rabbit semen have primarily focused on assessing the effects of antioxidant supplementation through diet [17,18] or extenders [14,15,19,20,21] and on comparing different storage conditions [22] rather than on characterizing intrinsic antioxidant responses during storage.

While OS is a major factor influencing sperm quality during chilled storage, genetic factors also play a significant role in determining semen storage potential. Differences in sperm cryotolerance have been reported between rabbit breeds [23,24] and even between individual males within the same breed [25,26]. However, studies examining breed-related variations in responses to chilling storage, especially in local rabbit populations, are lacking.

In Algeria, the local Algerian rabbit population (LAP) is an important genetic resource that has received little research attention regarding male reproduction. Most research has evaluated only fresh semen parameters, but not acrosome status, OS biomarkers, or cold storage resistance [27,28,29,30,31,32].

To address these gaps, this study aimed to characterize the resistance of LAP rabbit semen to chilled storage at 5 °C over 72 h, in comparison to the international standard well-documented NZW breed. We assessed conventional sperm quality parameters, acrosome status, and oxidative stress biomarkers in SP. Specifically, this study sought to evaluate semen preservation potential across breeds and to explore the relationship between sperm quality decline and OS biomarkers during storage regardless of breed. These findings may inform the development of optimized preservation protocols and improve the efficiency of AI programs, both in Algeria and globally.

## 2. Materials and Methods

### 2.1. Animals and Management

A total of 16 mature (24-month-old) and clinically healthy rabbit bucks were involved in the study, 8 bucks from the local Algerian population (LAP: 3178 ± 235 g) and 8 from the New Zealand White breed (NZW: 3408 ± 268 g). Animals were individually housed in flat-deck galvanized wire cages equipped with feed hoppers and an automatic nipple watering system. The present experiment was conducted during the winter season and lasted for 9 weeks (January–March). The lighting was natural, and the diurnal room temperature and relative humidity were on average 14.9 ± 2.5 °C and 71.0 ± 7.1%, respectively. Rabbits were fed a commercial diet (dry matter 90.7%, crude protein 13.3%, crude fat 2.6%, crude fiber 16%, and minerals 7.6%) and were provided with food and water ad libitum.

### 2.2. Semen Collection and Initial Evaluation

All rabbit bucks were previously trained for the semen collection procedure using an artificial vagina and a teaser doe. Semen was collected once weekly in the morning using a lubricated and pre-warmed artificial vagina. Immediately after collection, if present, the gel plug was removed, and pH (pH meter) and volume (conical graduated tube) of ejaculates were measured. The ejaculates were placed in a water bath and maintained at 37 °C until evaluation. Each ejaculate was assessed microscopically for initial semen quality, including mass motility (MM: scale of 0 to 9), individual motility (IM: scale from 0 to 4), motility percentage, live spermatozoa percentage, and sperm cell concentration (Thomas cell counter after semen dilution 1:200), according to the methods described by Boussit [33]. Only ejaculates with a standard color (not contaminated by urine or blood), a volume higher than 0.2 mL, a mass motility score over 6 (good wave motion), an individual motility score of 3 or 4 (linear movement), and more than 70% motility, 75% viability, and 300 million spermatozoa/mL were included in this study.

### 2.3. Semen Processing

Qualified ejaculates were pooled to avoid individual differences within each rabbit breed (6–8 ejaculates/pool; 9 pools/rabbit breed). A portion from each pool was removed in order to evaluate concentration, sperm motility, viability, and acrosome status. Then, each pool of semen was diluted to a final concentration of approximately 50 × 10^6^ sperms/mL with a Tris–citric acid–glucose liquid extender (250.04 mM TRIS, 79.76 mM citric acid.H_2_O, 69.38 mM glucose, 75.00 IU streptomycin, and 166.20 IUG–Penicillin, pH 7.14 and osmolality 299 mOsm/kg) [34]. To avoid thermal shock, equal volumes (20 mL) of extended semen samples were preserved under refrigeration (4–5 °C) in sterile Falcon tubes immersed in a glass beaker with pre-warmed water (37 °C) in order to reduce the temperature gradually, and, after 4 h, the tubes were taken out of the beaker and placed horizontally to prevent sperm pellet formation in the tip of the tube [35]. A semen sample was taken out from freshly diluted semen (0 h) and, at 4, 24, 48, and 72 h post-chilling, warmed to 37 °C for 5 min and then checked for semen quality, acrosome status, and SP oxidative stress parameters.

### 2.4. Assessment of Semen Quality

#### 2.4.1. Sperm Motility

Sperm motility was evaluated by visual estimation using a microscope equipped with a heated stage maintained at 37 °C (Olympus CX21FS2: Olympus Corporation, Tokyo, Japan) [33]. First, a drop of raw semen was placed on a clean, pre-warmed glass slide and observed at ×100 magnification to assess mass motility, which was scored based on the intensity of wave-like movement using a scale from 0 (no sperm movement) to 9 (strong, vigorous waves with a whirlwind appearance). Then, a drop of diluted semen from the same sample was placed on another clean, pre-warmed slide, covered with a coverslip, and examined at ×400 magnification. Under these conditions, both individual motility (scored from 0: immotile sperm to 4: fast, progressive, linear movement) and the percentage of motile spermatozoa (sperm showing any type of movement) were evaluated across several microscopic fields. The average of these observations was recorded as the final motility score for each semen sample.

#### 2.4.2. Sperm Viability (Membrane Integrity)

Sperm membrane integrity was evaluated using the eosin Y (1%) and nigrosin (10%) staining protocol as a viability indicator [36]. Briefly, 10 µL of semen and 20 µL of eosine–nigrosin stain were mixed gently at 37 °C and then smeared. Immediately after the smear had dried, at least 200 spermatozoa were examined in each sample microscopically at x400 magnification. Spermatozoa showing a partial or complete dark pink-stained head, indicating loss of membrane integrity, were considered non-viable, whereas only spermatozoa showing no incorporation of the stain were considered to be viable.

### 2.5. Assessment of Acrosome Status

The acrosome state of rabbit sperm was evaluated using a chlortetracycline (CTC) fluorescence assay, as previously described by Kaul et al. [37]. The CTC staining solution was prepared by dissolving 750 µM CTC in a buffer containing 20 mM Tris–HCl, 130 mM NaCl, and 5 mM cysteine pH 7.0. The fresh CTC staining solution was protected from light and was prepared for use on the same day. An aliquot of semen (20 µL) was mixed with 20 µL CTC stock and immediately fixed by adding 4 µL 12.5% (*w*/*v*) glutaraldehyde in 0.5M Tris–HCl (pH 7.4). Slides were prepared by mixing 10 µL of the fixed solution with one drop of antifade (0.22M DABCO dissolved in glycerol/PBS). A coverslip was placed on top of the slides, sealed with colorless nail varnish, and kept in the dark at 4 °C until the analysis was conducted within 24 h. At least 200 sperm per slide were evaluated under an epifluorescence microscope at ×1000 (Zeiss Axio Scope A1; Excitation filter 450–490 nm: Carl Zeiss, Oberkochen, Germany). Three distinct sperm fluorescence patterns were observed [38]: bright fluorescence over the sperm head, indicating an un-capacitated acrosome (IN); a non-fluorescent band in the post-acrosomal region of the sperm head, typical of a capacitated acrosome (CP); and weak or absent fluorescence observed over the sperm head with a bright equatorial band sometimes present, which is characteristic of capacitated acrosome-reacted cells (AR).

### 2.6. Assessment of Oxidative Stress Biomarkers in Seminal Plasma

Semen samples were taken out at 0, 4, 24, 48, and 72 h post-chilling and centrifuged for 15 min at 5000 rpm in order to separate the cells from the diluted SP. A drop of the supernatant composed of semen extender and SP was evaluated by microscope to confirm the absence of cells, and sperm-free SP was stored at −80 °C until further analysis. All SP analysis were performed using a Shimadzu U spectrophotometer (Shimadzu Corporation, Kyoto, Japan).

#### 2.6.1. Reactive Oxygen Metabolites (ROMs)

Oxidative status was evaluated by measuring reactive oxygen metabolites (hydroperoxides primarily) in the samples using the d-ROM test (Diacron International, Grosseto, Italy). The test is based on the principle that the amount of organic hydroperoxides present in the sample is related to the free radicals from which they are formed. ROMs concentration was determined spectrophotometrically following the manufacturer’s protocol (absorbance at 505 nm) and expressed as mg H_2_O_2_/dL.

#### 2.6.2. Lipid Peroxidation (LPO) Assay

LPO level was assessed by measuring malondialdehyde (MDA) production using thiobarbituric acid (TBA), according to the method described by Ohkawa et al. [39]. An aliquot of 100 µL of extended SP was added to a reaction mixture containing 50 mL of 8.1% sodium dodecyl sulfate solution, 375 mL of 20% acetic acid solution (pH 3.5), and 375 mL of 0.8% TBA solution. The mixture was then heated at 95 °C for 1 h. The samples were cooled and centrifuged at 3000× *g* for 10 min, and the absorbance of the supernatant was measured at 532 nm. The MDA concentration was calculated using the molar extinction coefficient (ε = 1.56 × 10^5^ mmol/L/cm) and was expressed as nmol MDA content/mL.

#### 2.6.3. Total Superoxide Dismutase (SOD) Activity

SOD activity was measured using the pyrogallol autoxidation assay, which is based on the ability of SOD to inhibit the autoxidation of pyrogallol at alkaline pH [40]. An amount of 10 µL of pyrogallol (a final concentration of 0.3 mM) was added to cuvettes containing 0,9 mL of reaction buffer (50 mM cacodylic acid, 1 mM DTPA, pH 8.2) and 0,1 mL of sample. Reaction was initiated by the addition of pyrogallol. After mixing, the reaction was monitored spectrophotometrically at 420 nm every 15 s for 5 min. The decrease in the autoxidation rate of control (sample-free) is related to the activity of SOD present in the sample. Enzyme activity was expressed as U of SOD/mL. One unit of SOD is defined as the amount of the enzyme required to inhibit 50% of pyrogallol autooxidation [40].

#### 2.6.4. Glutathione Peroxidase (GPX) Activity

GPX activity was measured by the evaluation of GSH oxidation rate by H_2_O_2_ following the method of Flohe and Gunzler [41]. A reaction mixture of 0.2 mL of GSH (2 mM), 0.3 mL of phosphate buffer (0.1 M, pH 7.4), 0.1 mL of sodium azide (10 mM), and 0.1 mL of hydrogen peroxide (1 mM) was added to 0.3 mL of the sample and incubated at 37 °C for 15 min. The reaction was stopped by the addition of 0.5 mL of 5% TCA and the mixture was centrifuged at 3000× *g* for 10 min. Subsequently, 0.1 mL of the obtained supernatant was added to 0.2 mL of phosphate buffer (0.1 M, pH 7.4) and 0.7 mL of 5,5′ dithio-bis-(2-nitrobenzoic acid) [DTNB (0.4 mg/mL)]. After mixing, absorbance was recorded spectrophotometrically at 420 nm. Enzyme activity was expressed as µmol GSH oxidized/L.

#### 2.6.5. Catalase (CAT) Activity

Catalase activity was determined using a colorimetric method based on the reduction of dichromate in acetic acid to chromic acetate when heated in the presence of H_2_O_2_. During this reaction, perchromic acid is formed as an unstable intermediate. The amount of chromic acetate produced is directly proportional to the concentration of H_2_O_2_ remaining [42]. An amount of 1.0 mL of 65 mM H_2_O_2_ in sodium and potassium phosphate buffer (50 mM, pH 7.4) was added to 100 µL of sample. The reaction (incubation at 37 °C) was stopped after three minutes by adding 2 mL of dichromate–acetic acid mixture. The tubes were kept at 100 °C for 10 min and then cooled, and the color developed was read at 570 nm against the reagent blank. Catalase activity was calculated using the first-order reaction rate constant k based on the decrease in H_2_O_2_ concentration (decomposition of H_2_O_2_ by catalase) over the enzymatic reaction time and expressed as kU/mL [42].

### 2.7. Statistical Analysis

Data were statistically analyzed using the lme4 packages of R software (version 4.1.1) (R Development Core Team, 2021) and are shown as mean ± standard error of the mean (SEM). First, data were tested for normality using QQ-plots and then non-normal data were transformed using log or arcsine. Subsequently, the impact of rabbit breed (two levels), storage time (five levels), and their interaction on the semen quality traits, acrosome status, and OS biomarkers was tested using a Generalized Linear Mixed Model (GLMM), with repeated measures by pool as a random effect. If the impact of storage time was statistically significant, multiple pair-wise comparisons between levels using the post hoc Tukey test were performed. Residuals of the model were checked for normality and homoscedasticity by examining descriptive statistics and plots. Finally, overall correlation among the measured parameters was evaluated using Spearman’s correlation coefficient, without taking into consideration breed effects and aggregating different time points into a single pooled dataset. Statistical significance was considered at *p* < 0.05.

## 3. Results

### 3.1. Assessment of Freshly Pooled Semen Quality

Before dilution, LAP and NZW rabbit bucks displayed similar semen quality traits, except for pH and pooled semen concentration (*p* < 0.05, Table 1).

### 3.2. Assessment of Semen Quality During Chilling Storage

Since the interaction between breed and storage time was not statistically significant for any of the traits analyzed, only the main effects are presented and discussed in the following sections.

#### 3.2.1. Sperm Quality: Motility and Viability

**Figure 1:**
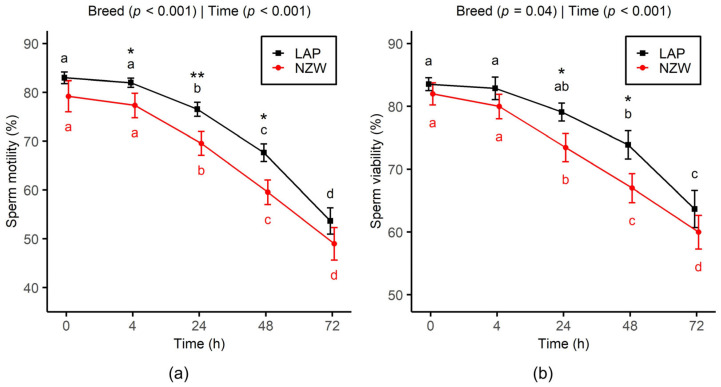
Effect of chilled storage (0, 4, 24, 48, and 72 h) on sperm motility (**a**) and viability (**b**) of LAP and NZW rabbits. Data are shown as the mean ± SEM. Different lowercase letters (a, b, c, and d) indicate significant differences (*p* < 0.05) between storage time points within the same breed. Asterisks indicate significant differences between breeds at the same time point (* *p* < 0.05 and ** *p* < 0.01). *p*-values from GLMM analysis for breed and storage time are displayed directly on each plot.

A steady significant decline in both sperm motility and viability was observed in LAP and NZW rabbit bucks with increasing chilling storage time (*p *< 0.001). Regarding sperm motility (Figure 1a), values remained relatively stable up to 4 h, followed by a significant decline after 24 h of cooling. A drop of 10% in motility was observed from 0 h to 24 h (*p* < 0.001), followed by a 30% drop from 24 h to 72 h (*p *< 0.001). In addition, from 4h to 48h, LAP semen exhibited significantly higher motility values than NZW (*p* < 0.05).

A significant decline in sperm viability was observed over the storage period from 0 to 72 h (*p* < 0.001), with an average decrease of -25.5% across both breeds (Figure 1b). However, a notable difference between the two breeds was detected, as membrane integrity in the NZW group significantly declined starting at 24 h, whereas a similar decrease was not observed in the LAP group until 48 h. Moreover, the LAP semen exhibited significantly higher viability between 24 h (*p =* 0.01) and 48 h (*p =* 0.03) of chilling storage.

#### 3.2.2. Acrosome Status

**Figure 2:**
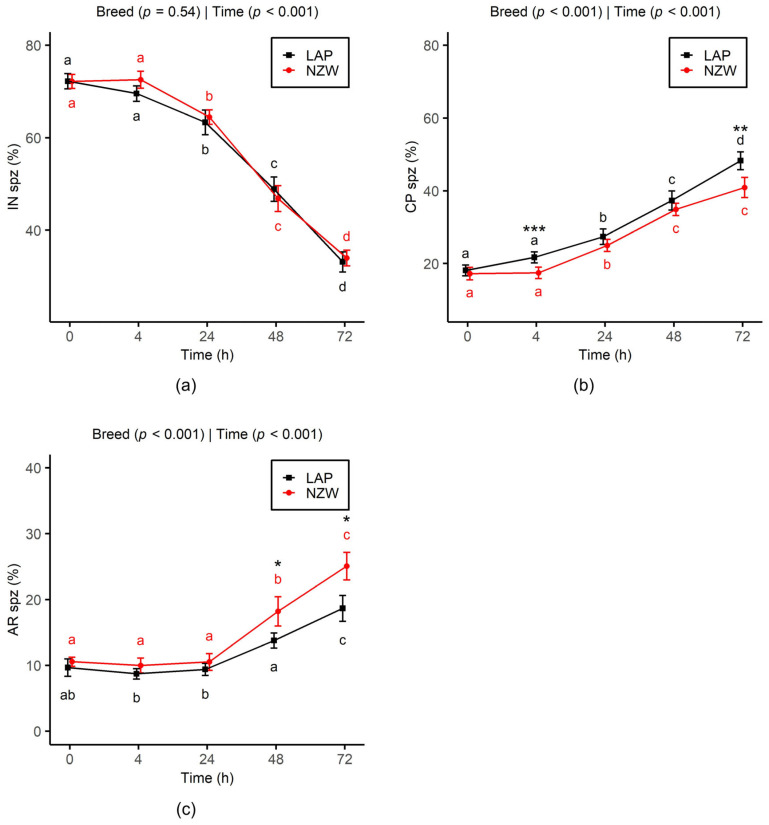
Effect of chilled storage (0, 4, 24, 48, and 72 h) on acrosome status ((**a**): un-capacitated acrosome (IN); (**b**): capacitated acrosome (CP); and (**c**): capacitated acrosome-reacted (AR)) of LAP and NZW rabbit sperm. Data are shown as the mean ± SEM. Different lowercase letters (a, b, c, and d) indicate significant differences (*p* < 0.05) between storage time points within the same breed. Asterisks indicate significant differences between breeds at the same time point (* *p* < 0.05, ** *p* < 0.01, and *** *p* < 0.001). *p*-values from GLMM analysis for breed and storage time are displayed directly on each plot.

The time-dependent evolution curves of un-capacitated (IN) sperm showed a significant decline from 24 h onward (*p* < 0.001), with a similar overall trend between the two breeds across all time points up to 72 h (Figure 2a). At the same time, sperm underwent capacitation (CP: Figure 2b) and acrosome reaction (AR: Figure 2c). As observed for IN sperm frequency, both breeds exhibited a similar evolution of CP and AR frequencies over time. The percentage of sperm displaying the CP pattern increased progressively from 24 h, while the AR pattern began to increase from 48 h. Furthermore, the frequency of CP sperm was significantly higher in LAP sperm compared to NZW sperm at 4 h (*p* < 0.001) and 72 h (*p* < 0.01), whereas the AR frequency was significantly lower in LAP sperm at 48 h (*p* = 0.04) and 72 h (*p* = 0.01).

#### 3.2.3. Oxidative Stress Biomarkers

**Figure 3:**
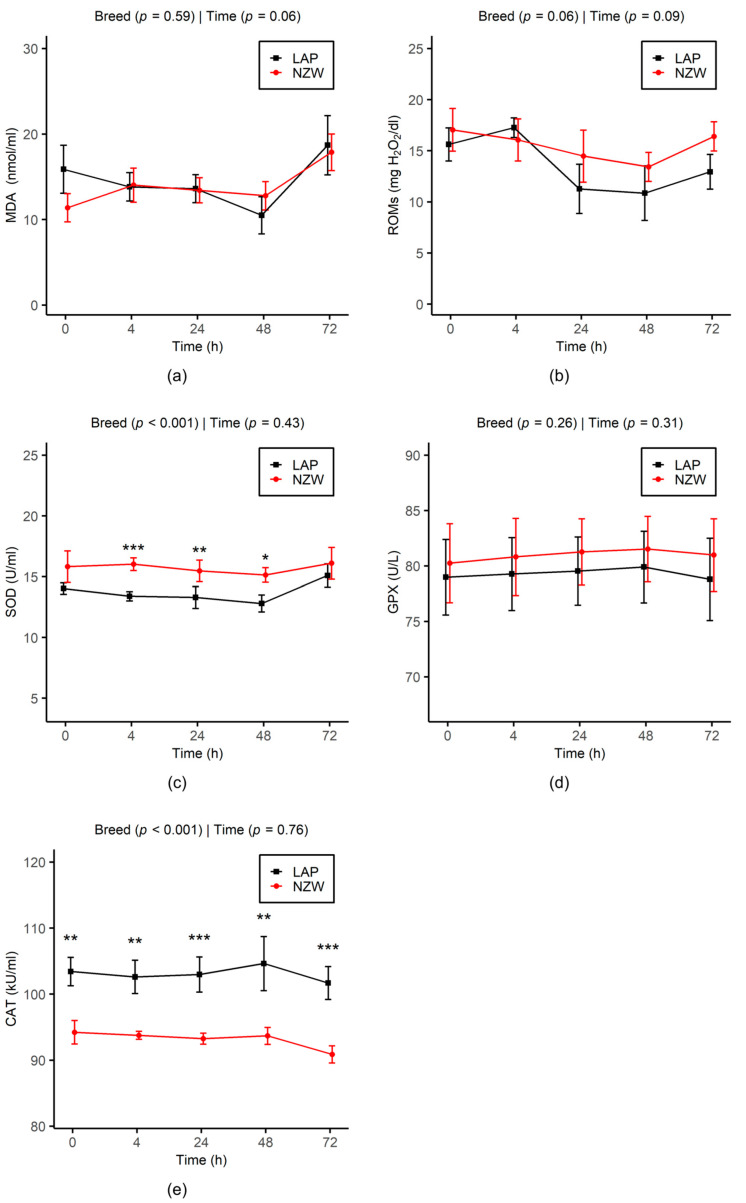
Effect of chilled storage (0, 4, 24, 48, and 72 h) on MDA (**a**) and ROMs (**b**) levels and SOD (**c**), GPX (**d**), and CAT (**e**) activities in seminal plasma of LAP and NZW rabbits. Data are shown as the mean ± SEM. Asterisks indicate significant differences between breeds at the same time point (* *p* < 0.05, ** *p* < 0.01, and *** *p* < 0.001). *p*-values from GLMM analysis for breed and storage time are displayed directly on each plot.

The effects of storage time and breed on the variation in SP oxidative stress parameters during chilling storage are reported in Figure 3.

In general, OS biomarkers in the SP remained consistent across the different time points analyzed for each breed group. Regarding the breed, no significant differences were observed for MDA, ROMs, and GPX between LAP and NZW buck rabbits. However, CAT activity remained significantly higher in the SP of LAP compared to NZW across all time points (*p* < 0.001). In contrast, SOD activity was significantly higher in NZW from 4 h (*p* < 0.001) to 48 h (*p* = 0.01).

### 3.3. Correlations Between Sperm Quality, Acrosome Status, and OS Markers

**Figure 4:**
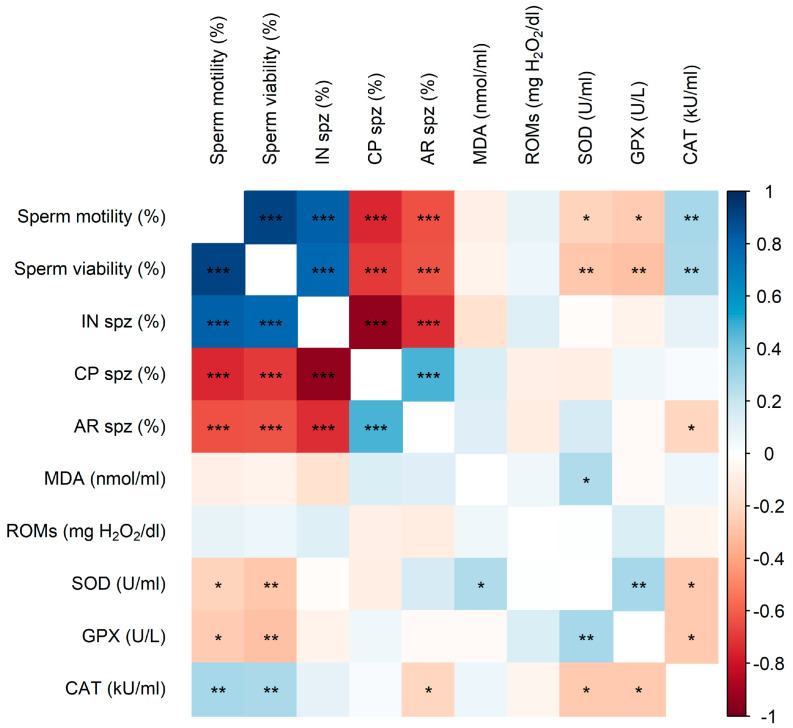
Heat map showing the correlations between sperm quality parameters (sperm motility and viability), acrosome status (IN, CP, and AR frequencies), and OS biomarkers in SP (MDA, ROMs, SOD, GPX, and CAT activities) of LAP and NZW rabbits. The colors on the scale (1 to −1) indicate whether the correlation is positive (blue) or negative (orange). (*) *p* < 0.05, (**) *p* < 0.01, and (***) *p* < 0.001.

Strong significant correlations (ρ *≥* |0.6|, *p* < 0.001) were observed among sperm quality and acrosome status parameters (Figure 4). Sperm motility, viability, and IN showed positive correlations with each other, while these parameters were negatively correlated with CP and AR.

Regarding the relationship between sperm quality, acrosome status on one side, and OS biomarkers on the other, weak but significant correlations *(*ρ ≤ |0.3|, *p* < 0.05) were found (Figure 4). Sperm motility and viability were negatively correlated with SOD and GPX activities but positively correlated with CAT activity. In contrast, AR was negatively correlated with CAT activity. Furthermore, SOD activity showed a significant positive correlation with both MDA levels and GPX activity, whereas CAT activity exhibited a significant negative correlation with both SOD and GPX.

## 4. Discussion

This study provides the first comparison between the LAP and the NZW rabbit bucks regarding sperm quality, acrosome status, and OS responses during chilled storage at 5 °C. Both breeds exhibited similar trends in semen quality, although LAP semen appeared more resilient to chilling deterioration, particularly between 4 and 48 h.

It is well known that semen production and sperm quality can vary across different breeds. Several studies have been carried out to explore this variability between local breeds and NZW bucks [43,44,45,46]. In the same context, our study demonstrated that LAP bucks exhibited lower fresh semen pH values compared to NZW bucks. This difference may be attributed to variations in SP composition, sperm concentration, and motility. The higher sperm concentration in LAP pools can lead to a more rapid decrease in pH due to the metabolic activity of the sperm, which releases lactic acid as a by-product [47,48]. Additionally, differences in pH may also result from the variation in accessory gland secretions between breeds [48].

As previously reported, LAP rabbits have a higher sperm concentration than NZW rabbits. This trait may be influenced by testicular size and testosterone levels, which have been linked to sperm concentration in other species [49,50]. In rabbits, sperm concentration was found to be highest in the small breed and lowest in the large breed [51]. Supporting this, some studies have observed that lighter-weight male rabbits have better sperm production parameters than heavier individuals [52,53].

Nevertheless, the majority of semen characteristics demonstrated no differences in semen production and sperm quality between the two rabbit breeds and indicate normal reproductive traits [54,55]. To the best of our knowledge, no prior studies have compared the semen of LAP rabbits to that of other exotic breeds. However, the limited available data on LAP rabbits, whether from similar or differing experimental conditions, indicate that the semen quality observed in our study is generally comparable or even superior to that reported in earlier studies on LAP rabbits [29,31,32]. Further work and a larger sample size are required to adequately characterize and compare the semen of LAP rabbit bucks to that of the well-known and widespread NZW rabbits.

Immediately after dilution at 0 h, the mean values of all studied sperm and seminal plasma parameters, except for CAT activity, showed no significant differences between the two rabbit breeds, indicating comparable baseline levels prior to chilling storage. However, as storage progressed, breed-specific differences emerged, despite a general decline in semen quality observed in both groups, consistent with previous findings in different rabbit breeds [5,15,18,21,56].

Sperm motility remains the basic parameter for predicting the fertility of rabbit sperm after semen storage, as it depends on both viability and structural integrity [57,58,59]. Previous studies in NZW rabbit bucks reported a similar decreasing trend in sperm motility and viability, although with varying degrees of intensity [5,17,21,56].

The comparison between the LAP and NZW rabbit bucks highlights some differences in terms of resistance to chilling storage. While both breeds generally follow similar trends regarding the evolution of sperm quality over time, the LAP bucks appear to be more resilient to the detrimental effects of chilling storage. They maintain higher sperm motility (from 4 to 48 h) and membrane integrity (from 24 to 48 h) compared to the NZW bucks. To the best of our knowledge, no studies have specifically investigated the differences between rabbit breeds concerning chilling semen preservation. However, variations in sperm resistance to cryopreservation are well-documented in the literature. Kulíková et al. [24] showed differences in sperm quality traits of fresh and frozen–thawed semen from four Slovak native rabbit breeds. Furthermore, comparisons of semen of different selected rabbit lines and studies of genetic correlations revealed that sperm from growth-selected lines exhibit lower quality and fertility following frozen–thawed procedures [23,60]. Our results seem to agree with these observations: the LAP population, which has not been selected for growth traits, showed better resistance to chilling storage compared to the more production-oriented NZW breed. Selection for growth traits in NZW may have altered sperm plasma membrane and/or SP composition, which are related to sperm cryo-resistance [60]. Several reports indicate associations between sperm cryo-resistance and the concentration of particular components in SP of some animal species [61,62,63]. In rabbit, the genetic origin influences the abundance of several sperm and seminal plasma proteins associated with sperm quality [64,65,66]. This could explain the differences in sperm mobility and membrane integrity observed in our study between LAP and NZW semen pools during chilling storage.

Our results regarding acrosome status indicated that LAP and NZW rabbit bucks showed a decline in frequencies of IN sperm with a similar rise in frequencies of CP and AR sperm over storage time. Research on the capacitation status of rabbit semen during chilled storage using CTC staining remains limited. However, our findings align closely with those of Castellini et al. [17], who reported that after 24h of chilling, NZW rabbit semen contained 60.4% IN, 21.3% CP, and 18.3% AR sperm (Cf. 64.5%, 25%, and 10.5%, respectively).

Notable differences in the progression of CP and AR suggest a specific breed response to storage. In particular, from 48 to 72 h of storage, NZW sperm exhibited a higher proportion of AR sperm. The phenomena observed during storage must be considered dynamic, given that they require a transition from intact to capacitated sperm and from capacitated sperm to acrosome-reacted sperm [38]. Thus, it is very intriguing and complex mechanism, but it is reasonable to hypothesize that the observed increase in AR sperm is a direct consequence of the earlier rise in CP sperm.

The differences in CP and AR dynamics observed between the LAP and NZW sperm may be explained by the composition of SP, particularly the abundance and/or composition of their seminal granules, which are known to work synergistically. As suggested by Castellini et al. [38], SP primarily exerts a de-capacitation effect, while seminal particles prevent CP sperm from undergoing premature AR.

Further investigation into the characterization of seminal particles could provide deeper insights into the mechanisms underlying the differences in sperm capacitation status between these two rabbit breeds during chilling storage.

One of the most important factors responsible for declining sperm quality during sperm processing and storage is OS. In our study, no variation in ROMs and MDA levels, key indicators of OS, were observed in SP of LAP and NZW rabbit bucks over 72 h of storage. This finding suggests that OS, and, in particular, LPO, is not a major factor influencing semen quality or acrosome status under our conditions and there might be other factors having a detrimental effect on the quality of stored semen. The levels of ROMs and MDA suggest that the semen’s natural defense against OS is either highly effective or not significantly challenged, i.e., low rate of RONS generation [67]. Our results are further supported by the stability of SOD, GPX, and CAT activities in the SP over 72 h of chilling storage in both breeds, suggesting that endogenous antioxidant enzymes effectively neutralized RONS and maintained an equilibrium between production and scavenging of radicals. The abundance of CAT in rabbit SP compared to other animals [68] makes rabbit sperm particularly efficient at eliminating H_2_O_2_ [69]. To the best of our knowledge, our study is the first to investigate the relationship between OS biomarkers in rabbit SP and semen characteristics during chilling storage at 5 °C. Previous studies have primarily focused on assessing the effects of antioxidants supplementation in the diet [17,18] or extenders [14,15,19,20,21] and on comparing different storage conditions [22]. Different results were reported under similar storage conditions. Some studies observed no changes in LPO, GPX, or SOD activity after 24 h and 72 h of storage [17,20,21]. In contrast, other studies found significant elevations in ROS and LPO after 72 h of chilling, with corresponding changes in antioxidant enzyme activities, such as SOD, CAT, and GPX [19,21,70].

Comparing the two rabbit breeds, no significant differences were observed in ROMs, MDA, and GPX. This indicates that LAP and NZW rabbits demonstrate comparable oxidative resistance within 72 h of chilling storage. It should be noted that GPX contributes to the elimination of H_2_O_2_ and also detoxifies reactive lipids by using glutathione (GSH) as a reducing agent. However, due to the low level of GSH in rabbit semen, the antioxidant role of GPX is considerably reduced compared to other species [71,72]. This implies that radical detoxification in rabbit semen relies primarily on SOD activity [73]. Moreover, as discussed earlier, rabbit semen has high CAT activity, which plays a key role in H_2_O_2_ neutralization, effectively complementing SOD activity in maintaining oxidative balance [68].

The SP of LAP rabbits showed markedly elevated CAT activity in fresh samples and during the storage period. This result aligns with findings by Casares-Crespo et al. [65], who reported differences in CAT expression between rabbit genetic lines, suggesting that higher catalase activity in SP may contribute to better post-thaw semen quality [23]. This would be in agreement with our results, as CAT activity in SP was positively related to the percentage of sperm motility and viability. Moreover, this genetic variation in CAT activity in fresh SP was also showed in four exotic rabbit breeds reared in Nigeria [74]. In line with this, the heritability of seminal catalase indicated that genetic factors contribute significantly to the observed variation among male rabbits [68].

Conversely, NZW semen showed higher SOD activity but only between 4 h and 48 h post-chilling. Elevated SOD activity in NZW rabbit fresh SP has been previously observed in comparison to Chinchilla rabbits [74]. In other species, Barranco et al. [75] reported that SOD activity in boar SP varies among individual animals, while it does not differ between breeds. Moreover, cooling for 24 h or 96 h prior to freezing has been shown to decrease SOD activity in frozen–thawed dog sperm [76] and treatments with SOD did not influence plasma membrane integrity in rabbit semen stored for up to 24 h [20]. The elevated SOD activity in NZW semen may, in turn, result in higher H_2_O_2_ accumulation, particularly connected with the lower CAT activity of this breed compared to LAP. Consequently, the greater dismutation of O_2_•− by SOD in NZW semen likely contributes to an increased presence of H_2_O_2_, which could be associated with the higher frequency of AR observed. This H_2_O_2_ presence may serve as a signaling pathway, stimulating CP sperm to undergo AR. Supporting this, Hsu et al. [77] observed that adding catalase inhibited the acrosome reaction, suggesting that H_2_O_2_ generated during capacitation plays a role in later stages of this process. In line with these findings, the significant negative correlation observed in our study between AR and CAT activity, the primary scavenger of H_2_O_2_ in SP, further supports the role of this ROS in mediating the AR.

The decrease in SOD activity during storage may also represent an adaptive response to limit H_2_O_2_ accumulation with the gradual rise in altered and dead sperm in both rabbit groups. Interestingly, the significant positive correlation between SOD and MDA in our study highlights how increased SOD activity, by generating H_2_O_2_, could contribute to LPO under chilling storage conditions. This interplay underscores the link between OS and SOD activity and supports the idea that the observed decrease in SOD activity during this period could help control ROS levels and limit oxidative damage. In fact, sperm are relatively resistant to the O_2_•− radical, which has a short lifespan and is relatively inert and unable to traverse the intact cell membrane. In contrast, they are highly vulnerable to H_2_O_2_, a reactive molecule that can permeate plasma membranes [78]. In support of this hypothesis, the higher SOD activity observed in the SP of NZW rabbits in our study coincided with a phase where sperm motility was unexpectedly lower than that of LAP sperm. Previous studies have shown that exposure of boar sperm to H_2_O_2_ for just 30 min was sufficient to induce a significant decline in motility, while plasma membrane integrity remained unaffected [67]. Furthermore, this decrease could also indicate a potential requirement for an optimal level of O_2_•− to function as a signaling molecule, thereby stimulating sperm motility, especially since it has been shown that sperm hyperactivation was associated with low SOD activity in SP and that extracellular O_2_•− was involved in hyperactivation in human spermatozoa [79].

It is worth noting that in the present study, a positive correlation was observed between SOD and GPX activities in SP, while both were negatively correlated with CAT activity. Although SOD and GPX are key antioxidant enzymes in rabbit SP, their activities have also been reported in rabbit sperm [17,73,80]. This suggests that the increase in SOD and GPX activity during chilling storage may, at least in part, be attributed to the leakage of intracellular enzymes from sperm cells to SP, as their activities were also negatively correlated with membrane integrity and motility. However, unlike SOD and GPX, rabbit sperm have weak intracellular CAT activity, with the majority of catalase in semen originating from SP [71,81]. Based on these findings, it could be suggested that in rabbit semen, SOD is primarily provided by sperm, whereas CAT is predominantly ensured by SP.

While this study highlights some breed-specific physiological and biochemical responses of rabbit semen to chilled storage, some limitations should be acknowledged. The absence of in vivo fertility trials limits the direct extrapolation of in vitro findings to actual reproductive performance following AI. Moreover, molecular analyses such as protein profiling and regulatory pathway exploration potentially involving genetic variants were beyond the scope of this work. Future studies integrating omics approaches are needed to elucidate the mechanisms underlying semen preservation and validate functional biomarkers.

## 5. Conclusions

This study demonstrates breed-dependent differences in rabbit semen responses to chilling storage. While fresh semen parameters were comparable, LAP bucks exhibited statistically enhanced sperm quality and superior acrosomal stability during chilling storage. These functional advantages were associated with a more balanced oxidative response: LAP semen exhibited elevated CAT activity, positively correlated with motility and viability and negatively correlated with AR. Conversely, they showed lower SOD activity, which correlated negatively with motility and viability and positively with oxidative traits.

These findings address our objectives by (1) characterizing semen responses to chilled storage, which revealed better resistance of LAP semen compared to NZW, and (2) identifying CAT and SOD activities in seminal plasma as potential biomarkers for assessing sperm quality decline during chilled storage. While these results provide a physiological basis for developing optimized semen preservation protocols, further studies incorporating fertility trials and molecular analyses are required to validate these results and elucidate the underlying mechanisms.

## Figures and Tables

**Table 1:** Quality traits of fresh semen collected from LAP and NZW rabbit bucks (mean ± SEM; N =  9).

Semen Variables	LAP	NWZ	*p*
pH	7.54 ± 0.03	7.61 ± 0.04	0.01
Pool volume (mL)	5.61 ± 0.39	5.52 ± 0.59	0.75
Pool concentration (10^6^ spz/mL)	688.44 ± 12.95	620.83 ± 29.85	0.03
Total sperm cells (10^6^ spz/pool)	3872.88 ± 296.18	3479.28 ± 476.42	0.11
MM (0–9)	9 ± 0	8.78 ± 0.22	0.29
IM (0–4)	4 ± 0	3.89 ± 0.11	0.30
Sperm motility (%)	84.44 ± 1	83.33 ± 1.67	0.52
Sperm viability (%)	85 ± 1.07	84 ± 1.47	0.55
IN spz (%)	74 ± 1.18	72.73 ± 1.2	0.56
CP spz (%)	19.33 ± 1.03	19 ± 1.52	0.85
AR spz (%)	6.67 ± 0.71	8.27 ± 0.87	0.20

Spz: spermatozoa, MM: mass motility, IM: individual motility, IN: un-capacitated acrosome, CP: capacitated acrosome, AR: acrosome-reacted.

## Data Availability

The data is contained within this article.
